# Decompression Tube Fixation on Adjacent Teeth for Cystic Lesions in the Jaws: A Long-Term Follow-Up Analysis of Predictive Factors

**DOI:** 10.7759/cureus.113177

**Published:** 2026-07-22

**Authors:** Akio Shibata, Kou Kawahara, Yutaro Kondo, Kumiko Hatsukawa, Yuki Tange

**Affiliations:** 1 Oral and Maxillofacial Surgery, Okazaki City Hospital, Okazaki, JPN; 2 Oral and Maxillofacial Surgery, Gifu Prefectural Tajimi Hospital, Tajimi, JPN; 3 Oral and Maxillofacial Surgery, Aichi Gakuin University, Nagoya, JPN

**Keywords:** conservative treatment, decompression, mandibular ramus, odontogenic cyst, reduction rate, wire tube fixation

## Abstract

Introduction

Decompression is an effective conservative treatment for large odontogenic cysts; however, conventional tube stabilization often encounters issues such as device dislodgement or mucosal trauma. This study aimed to analyze the therapeutic efficacy and clinical outcomes of a novel decompression tube fixation method secured directly to adjacent teeth using stainless steel wire.

Materials and methods

We retrospectively reviewed 20 patients (11 males, 9 females; mean age: 41.5 years) with jaw cysts treated between 2013 and 2023. Quantification of cyst dimensions and two-dimensional (2D) lesion area reduction rates (RR) was performed using panoramic radiographs and NIH Image software (National Institutes of Health, Bethesda, USA). The impact of clinical parameters - including lesion location, tube parameters (central vs. marginal placement), and a history of infection - on therapeutic efficacy was statistically analyzed.

Results

The cohort comprised radicular cysts (n=8, 40.0%), dentigerous cysts (n=7, 35.0%), odontogenic keratocysts (n=4, 20.0%), and a simple bone cyst (n=1, 5.0%). Outcomes were categorized as extremely effective (n=9, 45.0%), moderately effective (n=5, 25.0%), and poorly effective (n=6, 30.0%). Lesions in the mandibular body and maxilla achieved optimal results, whereas mandibular ramus lesions showed poorer responses. This location-dependent efficacy created an apparent trend toward better outcomes in elderly males, which was confounded by the predominance of ramus cases in younger females (<44 years). Centrally placed tubes (n=12, 60.0%) yielded more consistent treatment outcomes than marginally placed tubes(n=8, 40.0%), potentially due to a lower susceptibility to lumen occlusion by surrounding granulation tissue. A history of infection was associated with a lower RR. The mean decompression period was 17.6 months. A longitudinal analysis of individual patient data revealed distinct reduction trajectories at the six-month mark; patients with a >50% area RR at six months achieved a higher success rate (6/7 cases, 85.7%) compared to those with <50% reduction (6/13 cases, 46.1%)

Conclusions

Our method of decompression tube fixation onto adjacent teeth is a reliable, well-tolerated, and effective modality, even for older patients and large odontogenic cystic lesions. A six-month evaluation threshold (50% reduction) serves as an adequate clinical indicator for determining whether to continue decompression therapy or proceed to early definitive surgical enucleation.

## Introduction

Odontogenic cysts are lesions specific to the oral and maxillofacial region, arising from remnants of the odontogenic epithelium sequestered within the gingiva or jawbone [1, 2]. A variety of cysts frequently occur in the jaws, and over 90% of cysts in the oral and maxillofacial region are odontogenic in origin [2]. Decompression and marsupialization, originally proposed by Partsch in the late 19th century, remain fundamental therapeutic modalities [3].The procedure involves creating a surgical fenestration in the cystic wall to establish continuity with the oral cavity. By maintaining this patency with a drainage tube, internal cystic pressure is equalized with external atmospheric pressure, thereby facilitating spontaneous drainage and subsequent bone regeneration. Decompression has been employed as a more conservative therapeutic approach for large odontogenic cysts to minimize the cyst volume and a corresponding limitation in the magnitude of the surgical procedure [4]. This technique was described that intraluminal pressure can be reduced by creating an opening into the cystic cavity that is based on the rationale that releasing the intramural pressure causes the cysts to diminish by gradual bone growth from the periphery [4]. Several devices have been used successfully for decompression, as described in the international literature [5-7]. Some of the common problems encountered during the course of this type of treatment include the need for long-term monitoring, inappropriate decompression tube size, soft tissue trauma, suture dehiscence, soft tissue invagination, dislodgement, and malpositioning of the tube into the lesion. Although these are not major complications, they contribute to the discomfort and inconvenience for the patient and the clinician. Kolokythas et al. [8] described a method that involved using stainless steel ligature wire that secures the polyethylene tube around the tooth/teeth immediately adjacent to the site of the lesion. This method would solve these problems. However, questions remain regarding the effectiveness of decompression in reducing cyst size. However, there still remain some questions as to how effectively decompression can reduce the cyst size. The objective of this study was to clarify the clinical efficacy and safety of this novel tube fixation method. Specifically, we aimed to evaluate the long-term predictive values of three clinical factors: anatomical lesion location, tube placement parameters, and a history of prior infection. Furthermore, we tested the explicit hypothesis that a two-dimensional area reduction of >50% at the six-month mark could serve as a reliable preliminary threshold to guide clinician decision-making regarding the duration of decompression therapy

## Materials and methods

Subject screening

**Figure 1:**
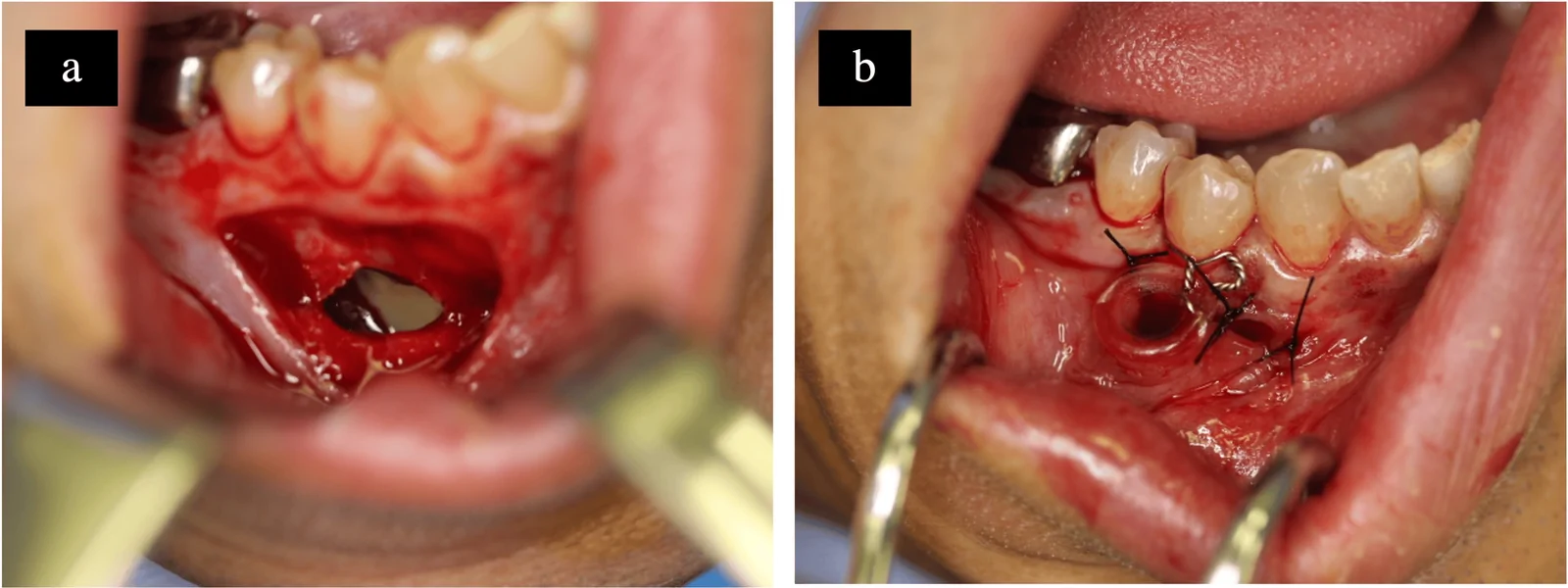
Decompression tube fixation on an adjacent tooth Decompression procedure performed concurrently with the incisional biopsy at the initiation of treatment.  (a) Intraoral findings during the incisional biopsy. (b) The decompression tube secured to the adjacent tooth using a 0.4-mm stainless steel wire. Source: Created by the authors using Microsoft PowerPoint (Microsoft Corporation, Redmond, USA) based on original institutional medical records.

We searched the medical records for cases where a device was used for decompression tube fixation on an adjacent tooth before surgery (Figure 1). We applied the decompression technique to a significant jaw cyst to minimize lesion volume and circumvent surgical complications arising from anatomical limitations. These constraints typically involve the inferior alveolar nerve and vessels, compromised cortical integrity, or engagement of the tooth apex. This strategy was specifically adopted to reduce the risk of recurrence associated with incomplete excision. Data were systematically collected across a comprehensive set of variables. These included patient age and gender, the lesion's location, radiolucency, locularity, and size, as well as the history of infection and the presence of unerupted teeth. Furthermore, the degree of lesion interference with adjacent anatomical structures was recorded, alongside parameters of the decompression therapy (tube position and depth, total duration), the initial histopathologic diagnosis, the resultant reduction rate (RR), the justification for definitive surgical intervention, and the postoperative follow-up period.

**Figure 2:**
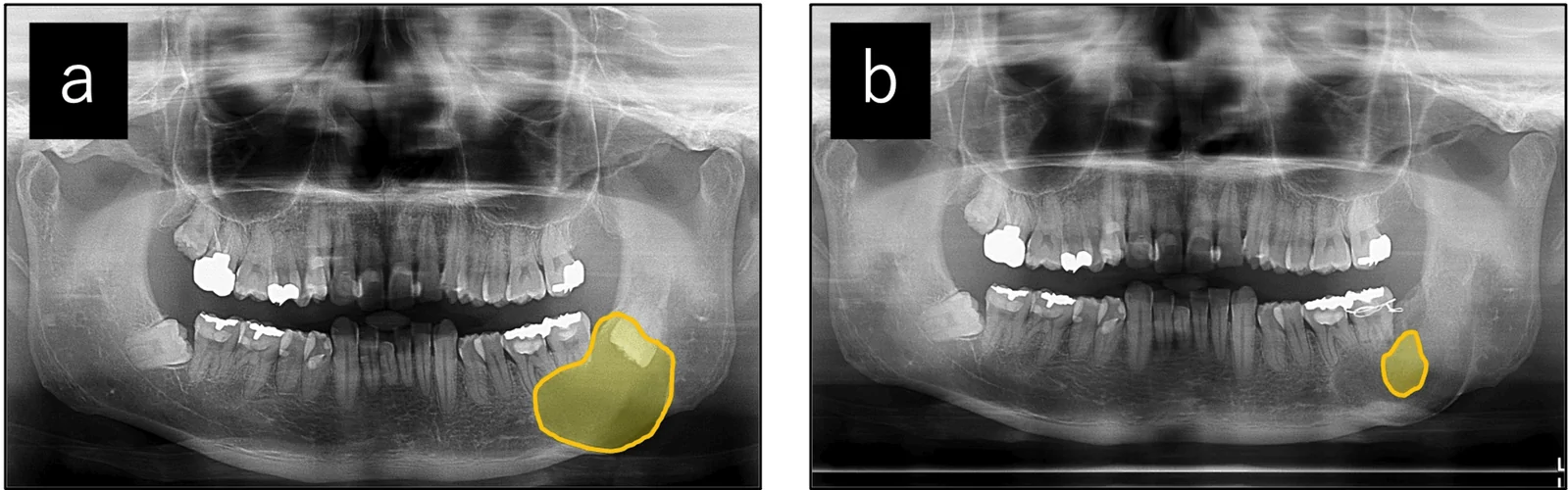
Measurement of lesion reduction rate using radiographic imagery. (a) Pre-decompression panoramic radiograph. (b) Post-decompression panoramic radiograph. The orange/yellow outlined and shaded areas represent the pre- and post-decompression lesion areas, respectively, which were segmented for pixel-based area quantification. Source: Patient radiographs obtained from the authors' clinical repository.

Evaluation of the decompression procedure's efficacy was achieved by scanning the pre- and post-decompression panoramic radiographic images at a standardized resolution of 100 pixels per inch. Quantification of the cyst dimensions was performed using the NIH Image software (National Institutes of Health, Bethesda, USA). The area of the cyst was determined by calculating the pixel count within the defined boundaries. Due to the inherent uneven magnification in the radiographic series, a correction factor was applied to the jaw dimensions. This involved equating the measured width of the first molar in the pre- and post-decompression films to ensure measurement consistency (Figure 2).

The reduction rate (RR) was calculated according to the method described by Nakamura et al. [6] using the following formula:

$Reduction Rate(\%) =\frac{(\text{Pre-decompression lesion area})-(\text{Post-decompression lesion area})}{(\text{Pre-decompression lesion area})}\mathrm{&times;100}$ 

The efficacy of the decompression was then graded based on the RR as follows: extremely effective (RR > 80%, including cases of complete cyst resolution during decompression), moderately effective (50% < RR ≤ 80%), and poorly effective (RR ≤ 50%). Exclusion criteria for the study were missing clinical or radiographic data, failure to maintain the decompression window, and follow-up of <6 months. All statistical analyses were performed using EZR (Saitama Medical Center, Jichi Medical University, Saitama, Japan), which is a graphical user interface for R (The R Foundation for Statistical Computing, Vienna, Austria) [9]. Statistical analysis was performed using the Mann-Whitney U test and the Pearson correlation coefficient. For the Mann-Whitney U test, statistical significance was established at a P-value of <0.05.

This retrospective study adhered to the principles of the Declaration of Helsinki. In accordance with the ethical guidelines for medical and health research involving human subjects in Japan, the requirement for direct written informed consent was waived. Alternatively, information regarding the study protocol, objectives, and data usage was publicly disclosed via an institutional opt-out method (hospital notice boards) to guarantee the patients' right to refuse participation. All personal, identifiable information was strictly anonymized prior to statistical analysis.

Decompression technique

Decompression was performed concurrently with the biopsy at the initiation of treatment. An opening was created, sized precisely to accommodate the incisional biopsy of the cystic lining, consistent with the technique described by Kolokythas et al. [8]. The procedure utilizes either a 16 Fr suction tube or a silicone tube equipped with a radiopaque line (Phycon tube, Fuji Systems Corporation, Tokyo, Japan). The tube is secured circumferentially around the tooth using a wire, and subsequently, a 0.4 mm stainless steel wire is threaded through the flange and twisted to fixate the tube. To prevent distal obstruction, the tube's end may be beveled (cut diagonally), or a fenestration (side hole) may be created, or both modifications may be applied [10]. The optimal tube length was determined via radiographic assessment of the lesion's extent. For multilocular lesions, intracystic septa were removed to establish a single cystic cavity. Following tube fixation, patients were comprehensively trained to irrigate the cystic cavity daily through the tube using sterile saline three times a day (strictly after each meal) to ensure patency and prevent food impaction. This regimen served to maintain the patency of the opening and prevent both food impaction and subsequent bacterial infection. Panoramic radiographs were obtained quarterly (every three months) for comparison with the baseline image. Cyst enucleation was then performed once two specific criteria were met: either when the lesion size had demonstrably ceased decreasing, or when the surgeon determined that the residual lesion allowed for the avoidance of iatrogenic damage to critical anatomical structures. During the definitive secondary enucleation of the four odontogenic keratocysts (OKCs), peripheral ostectomy was routinely performed as an adjunctive surgical measure to minimize the risk of recurrence.

## Results

This study included 20 cases with complete medical records collected during the period from April 2013 to March 2023 (Table 1). The follow-up period ranged from 11 to 64 months, with a mean duration of 27.8 months. The study included 20 patients (11 males (55.0%) and nine females (45.0%)) with an average age of 41.5 years. Regarding the anatomical distribution of the 20 lesions, 17 (85.0%) were located in the mandible and three (15.0%) in the maxilla. Mandibular involvement included 12 cases (60.0%) in the body and five cases (25.0%) in the region extending from the angle to the ramus. Radiographically, all lesions were unilocular, except for the simple bone cyst. The mean lesion length was 37.3 mm (range, 19-89 mm). A history of infection was present in seven cases (35.0%), and eight cases (40.0%) were associated with unerupted teeth. The criteria for selecting decompression were based on the following findings: lesion length >30 mm (n = 16, 80.0%); involvement of adjacent structures, including the mandibular canal (n = 17, 85.0%) and the maxillary sinus (n = 2, 10.0%); buccal and/or lingual cortical bone resorption (n = 10, 50.0%); and adjacent tooth involvement (n = 8, 40.0%). Preoperative histopathological evaluation of the 20 lesions revealed four distinct diagnoses: radicular cyst (RC; n = 8, 40.0%), dentigerous cyst (DC; n = 7, 35.0%), odontogenic keratocyst (OKC; n = 4, 20.0%), and simple bone cyst (SBC; n = 1, 5.0%). Regarding the treatment regimen, three patients (15.0%) were managed with decompression alone, while the majority (n = 17, 85.0%) underwent decompression followed by secondary enucleation. In all cases, the initial preoperative diagnosis was consistent with the final histopathological findings, with no diagnostic shifts observed following the decompression phase.

**Table 1:** Patient information ○: Resolved, ●: Unresolved, RR: Reduction Rate; IAN: Inferior Alveolar Nerve; M: Months

No.	Age	Gender	Location	Size（mm)		Tooth impaction	History of infection	Pathologic diagnosis	Tube position		Duration (M)	RR at 6 Months	Outcome	IAN	Bone defect	Contact tooth apex
1	18	F	Ramus	89	44	+	+	Odontogenic keratocyst	Marginal	Superficial	26	32%	moderately effective	○	○	-
2	19	F	Ramus	45	24	-	+	Odontogenic keratocyst	Marginal	Deep	21	28%	moderately effective	○	-	-
3	52	F	Body	35	16	-	-	Radicular cyst	Central	Superficial	7	90%	extremely effective	○	-	-
4	66	M	Body	33	9	-	-	Odontogenic keratocyst	Central	Deep	36	26%	extremely effective	○	-	-
5	66	M	Body	62	22	-	+	Odontogenic keratocyst	Marginal	Deep	12	30%	moderately effective	○	-	-
6	39	M	Body	37	14	+	-	Dentigerous cyst	Marginal	Superficial	22	27%	poorly effective	○	-	●
7	39	M	Body	21	11	-	+	Radicular cyst	Central	Deep	14	21%	poorly effective	○	-	-
8	46	M	Body	34	9	-	-	Radicular cyst	Central	Deep	6	42%	moderately effective	○	-	-
9	45	M	Body	36	17	+	-	Dentigerous cyst	Central	Deep	29	27%	extremely effective	○	○	-
10	67	F	Body	28	14	-	-	Radicular cyst	Central	Superficial	36	26%	poorly effective	○	●	-
11	46	M	Body	23	20	-	-	Radicular cyst	Central	Deep	6	81%	extremely effective	-	-	○
12	31	F	Body	19	10	-	+	Radicular cyst	Central	Deep	6	70%	moderately effective	○	○	●
13	32	M	Ramus	41	16	+	-	Dentigerous cyst	Marginal	Deep	16	46%	poorly effective	○	-	-
14	34	F	Body	30	16	+	-	Simple bone cyst	Marginal	Superficial	18	61%	extremely effective	○	○	○
15	53	M	Body	35	24	+	-	Dentigerous cyst	Marginal	Deep	36	33%	extremely effective	○	○	-
16	19	F	Ramus	32	16	+	-	Dentigerous cyst	Central	Deep	12	67%	extremely effective	○	○	-
17	14	F	Ramus	51	22	+	+	Dentigerous cyst	Marginal	Superficial	7	45%	poorly effective	○	○	○
18	53	M	Maxilla	30	24	-	-	Radicular cyst	Central	Superficial	13	55%	extremely effective	-	○	○
19	43	M	Maxilla	41	31	-	+	Dentigerous cyst	Central	Deep	10	55%	extremely effective	○	○	○
20	47	F	Maxilla	24	20	-	-	Radicular cyst	Central	Deep	18	28%	poorly effective	-	-	●

Regarding the decompression tube, placement was marginal in eight cases (40.0%) and central in 12 cases (60.0%), with a deep position used in 13 cases (65.0%) and a superficial position in seven cases (35.0%). The mean decompression period was 17.6 months (range, 6-36 months). Based on the lesion RR, outcomes were categorized as extremely effective (n = 9, 45.0%), moderately effective (n = 5, 25.0%), and poorly effective (n = 6, 30.0%). Resolution of anatomical constraints was achieved in 85.0% of cases. Specifically, the lesion was successfully separated from the inferior alveolar neurovascular bundle in 17 patients (85.0%), and cortical bone regeneration was observed within previous defect sites in 10 patients (50.0%). Furthermore, the lesion was released from the adjacent tooth apex in eight patients (40.0%). Overall, anatomical limitations were resolved in nearly all cases, with the exception of three patients in whom complete separation from the involved tooth apex could not be achieved. Adverse events were observed in two cases: one instance of tube dislodgment and one instance of bleeding from the fenestration site.

Subsequently, a comparative analysis of decompression outcomes was performed across various parameters. There was a weak, statistically non-significant positive correlation between lesion RR and decompression duration (Pearson’s r = 0.016). Notably, 85.7% (6/7 cases) of patients who exhibited a reduction of over 50% within the first six months were ultimately categorized into the "extremely effective" group. Conversely, the "poorly effective" group-characterized by prolonged decompression with less than 50% reduction at the six-month mark-accounted for 46.1% (6/13 cases) of the remaining patients. Regarding gender distribution, most cases in the "extremely effective" group were male (n = 6, 66.7%).

The mean ages for the extremely, moderately, and poorly effective groups were 45.7, 36.0, and 39.7 years, respectively, with no significant correlation between age and therapeutic efficacy. Regarding lesion location, those in the mandibular body achieved optimal results, whereas lesions extending from the mandibular angle to the ramus demonstrated a significantly poorer response, predominantly falling into the moderate or poor effectiveness categories (Table 2).

**Table 2:** Patient demographics and lesion characteristics by therapeutic effect Extremely effective: RR > 80%; Moderately effective: RR >50% and <80%; Poorly effective: RR < 50%.  Body: mandible body region; Ramus: mandible ramus; y: years old

Effect	Total Number	Sex		Age (y)			Location		
		Male	Female	Average	y≦44	44＜y	Body	Ramus	Maxilla
Extremely effective	9	6 (66.7%)	3 (33.3%)	45.7	2 (22.2%)	7 (77.8%)	6 (66.7%)	1 (11.1%)	2 (22.2%)
Moderately effective	5	2 (40.0%)	3 (60.0%)	36.0	3 (60.0%)	2 (40.0%)	3 (60.0%)	2 (40.0%)	-
Poorly effective	6	3 (50.0%)	3 (50.0%)	39.7	4 (66.7%)	2 (33.3%)	3 (50.0%)	2 (33.3%)	1 (17.7%)

Unerupted teeth did not influence the observed correlation. Conversely, a history of infection was associated with a lower RR compared to cases without such a history. While the RR did not differ significantly across histopathological diagnoses (DC, RC, OKC, and SBC), the resolution of anatomical constraints varied markedly. Complete resolution was achieved in all DC cases (7/7, 100.0%) and most RC cases (7/8, 87.5%), but only in 50.0% of OKC cases (2/4). Regarding tube parameters, marginal placement (n=8) resulted in an extremely effective reduction in 50.0% of cases, compared to 41.7% for central placement (n=12). Similarly, a deep tube position (n=13) showed a higher rate of "extremely effective" results (53.8%) than a superficial position (n=7, 28.6%). Despite these trends, statistical analysis indicated no significant correlation between tube parameters (position or depth) and decompression efficacy (Table 3).

**Table 3:** Distribution of therapeutic outcomes by clinical parameters: unerupted teeth, prior infection, and tube positioning Extremely effective: Reduction Rate (RR) > 80%; Moderately effective: RR >50% and <80%; Poorly effective: RR < 50%.

Effect	Total number	Unerupted teeth		Infection		Tube position		Tube depth	
		Yes	No	Yes	No	Central	Marginal	Deep	Superficial
Extremely effective	9	4 (44.4%)	5 (55.6%)	1 (11.1%)	8 (88.9%)	7 (77.8%)	2 (22.2%)	6 (66.7%)	3 (33.3%)
Moderately effective	5	1 (20.0%)	4 (80.0%)	4 (80.0%)	1 (20.0%)	2 (40.0%)	3 (60.0%)	4 (80.0%)	1 (20.0%)
Poorly effective	6	3 (50.0%)	3 (50.0%)	2 (33.3%)	4 (66.7%)	3 (50.0%)	3 (50.0%)	3 (50.0%)	3 (50.0%)

## Discussion

Marsupialization and decompression offer several key advantages: they induce a gradual decrease in the cystic cavity while preserving oral tissues and maintaining pulp vitality, preventing the need for dental extractions and the risk of iatrogenic surgical damage to critical anatomical structures, including the inferior alveolar nerve, maxillary sinus, nasal cavity, and developing teeth [4, 11]. The procedures also minimize the risk of mandibular fracture and are associated with a low rate of recurrence [4]. To optimize this conservative approach, our cases utilized a novel method of securing the decompression tube directly to the adjacent tooth. Since decompression was introduced as a conservative treatment for odontogenic cysts, numerous cases have been treated with this modality, with various studies reporting high success rates [6, 12, 13].

According to Tolstunov [14], an ideal decompression device should possess several key characteristics: it should be designed to prevent dislodgement into or out of the bone cavity during or after the procedure, be small enough not to interfere with daily mastication, and be easily fixed to the surrounding soft tissue. Furthermore, it must allow for easy daily irrigation of the cystic cavity through its opening by the patient or staff and be hygienic, ensuring it does not accumulate food particles while in use. Fixation of the decompression tube to the adjacent tooth effectively addresses these requirements and is well tolerated by patients; furthermore, it prevents mucosal or gingival trauma caused by micromovement or suture irritation [8].

Previous studies have demonstrated that several clinical factors, including patient sex, initial lesion size, anatomical location, and histopathological diagnosis, can significantly influence the therapeutic efficacy and reduction rate of decompression therapy [4, 11, 15]. Although decompression therapy is generally considered more effective in younger patients [4], our cases demonstrated a tendency toward a more favorable therapeutic response in elderly male patients. This discrepancy may be attributed to the anatomical distribution of the lesions; specifically, the ramus was involved in only one male patient compared to four female patients, and all five ramus cases occurred in patients under 44 years of age. Consequently, the apparent lack of efficacy in certain demographics may have been confounded by the anatomical location, which is inherently less responsive to decompression.

Placing the decompression tube at the center of the lesion yields more consistent treatment efficacy compared to peripheral placement. The inadequate decompression effect observed with marginal tube placement is likely attributed to several factors: the formation of an undercut or a closed lumen as the lesion contracts away from the insertion site, or the occlusion of the area around the insertion site by granulation tissue growth. In our study, for lesions in the mandibular body and maxilla, the tube was inserted centrally in 11 cases and peripherally in four cases. Conversely, the mandibular ramus showed the opposite distribution, with only one central placement compared to four peripheral placements. It is presumed that the greater number of cases with a centrally positioned tube in the mandibular body and maxilla contributed to the better treatment outcomes observed in those areas. Therefore, alternative marsupialization or decompression methods should be considered for cases where central tube placement is geometrically or anatomically unfeasible.

A trend was also observed in which cases with a prior history of infection achieved less favorable treatment outcomes. This clinical observation is consistent with previous studies showing that inflammatory or previously infected cysts, such as radicular cysts, exhibit significantly lower reduction rates compared to non-infected developmental cysts [11]. It was hypothesized that the reduced effectiveness of decompression therapy in these cases could be attributed to factors such as post-infectious scar fibrosis in the cyst wall and blood flow impairment resulting from the sclerotic hardening of the adjacent bone. Histopathologically, chronic inflammation and infection are known to induce severe collagen fibrosis and marked thickening of the fibrous capsule [16], which inherently restricts the elastic contraction and centripetal regression of the cystic lumen. Furthermore, reactive osteosclerosis (condensing osteitis) in the surrounding bone severely compromises local microvascularity, thereby suppressing the osteogenic potential necessary for bone neoformation after intracystic pressure is relieved.

Clinical outcomes often vary; some cases show early regression, while others demonstrate limited shrinkage even over an extended period. In support of this, Song et al. [15] analyzed the chronological volume reduction pattern after decompression and reported that the reduction rate is typically highest during the initial phases of treatment and gradually decelerates over time. For cases with poor therapeutic effect, it is difficult to relieve the anatomical constraints, meaning decompression therapy should not be continued indefinitely; instead, it is necessary to consider the timing of surgical enucleation earlier. Regarding the timing for determining the efficacy of treatment, our data suggest that if the size of the lesion has reduced by over 50% at 6 months, further reduction can be expected, and continued decompression should be considered. On the other hand, if the reduction is less than 50% at 6 months, the treatment is unlikely to be effective even after a prolonged period, and definitive surgical enucleation should be promptly considered. However, this proposed 6-month/50% reduction threshold should be interpreted as a preliminary, hypothesis-generating finding; while clinically instructive for this cohort, it requires further large-scale prospective validation to confirm its universal applicability. The findings of this study suggest potential trends regarding the outcomes of decompression therapy and three key factors: the location of the lesion, the placement of the tube, and the patient's history of infection. Our exploratory data indicate that less favorable results may be associated with mandibular ramus lesions, marginally positioned tubes, and patients who have experienced previous infection. The clinical variations in decompression effectiveness observed in this study may stem from anatomical and physiological factors. For instance, a history of infection induces dense intracystic fibrosis and bony sclerosis, which inherently resist rapid bony remodeling. Clinicians may preliminarily predict the effectiveness prior to tube placement by carefully evaluating cortical bone thickness, baseline multilocularity, and proximity to major anatomical structures via preoperative CT scanning. Consequently, understanding these predictive factors allows clinicians to optimize the application of this novel tube fixation method. By combining careful preoperative assessment with our stable adjacent-teeth fixation technique, the overall clinical approach becomes highly effective and well-tolerated, ultimately facilitating timely, minimally invasive secondary interventions.

Limitations

There are several limitations to this study. First, given our small sample size (N=20), drawing definitive conclusions regarding tight clinical correlations remains challenging, and the results should be viewed as exploratory. Second, another significant limitation is the reliance on two-dimensional panoramic radiographic area measurements rather than three-dimensional (3D) cone-beam computed tomography volumetric analyses. Panoramic imaging involves inherent geometric magnification and distortion, which may introduce minor measurement biases compared to 3D volumetric tracking.

## Conclusions

The usefulness of our method of decompression tube fixation was demonstrated even in older patients, particularly for large odontogenic cystic lesions such as dentigerous cysts. This technique provides a highly stable, patient-friendly alternative that minimizes technical complications associated with conventional tube retention. By optimizing patient compliance and maintaining reliable cavity patency, clinicians can achieve predictable cyst regression. A treatment period of at least six months appears adequate to determine whether to continue decompression therapy or proceed to definitive surgical intervention, as decompression has not yet been established as a definitive monotherapy. This clear evaluation window helps avoid unnecessarily prolonged treatment and facilitates timely, minimally invasive secondary enucleation. While these preliminary outcomes are promising, further multi-center studies with larger sample sizes are required before these predictive factors and thresholds can be universally generalized to standard clinical practice.
